# Iodide of Potassium in the Treatment of Infantile Broncho-Pneumonia

**Published:** 1886-08

**Authors:** 


IODIDE OF POTASSIUM IN THE TREATMENT OF INFANTILE BRONCHO-PNEUMONIA.—Dr. Zinnis, of Athens, Greece, says that potassium iodide in the broncho-pneumonia of children, from one to five years of age, especially in the sub-acute form, as nearly approaches a specific as can be. It is most useful in the early stages. He says it lowers the temperature, reduces the frequency of respirations and improves the local conditions rapidly. It is given in doses of eight to twenty grains, according to age, three times daily.—*N. Y. Medical Journal.*

Shpolanski has recently made a series of experiments on nineteen persons, to ascertain the time required for the digestion of milk and other foods under various conditions. Fresh milk remained in the stomach two hours and thirty minutes. On an average of seventeen observations, boiled milk, however, is digested in two hours and ten minutes. In a case of gastric catarrh, the time required was four hours, and in a case of chronic catarrh, complicated with dilatation, the milk remained in the stomach five hours and forty-five minutes. The author also finds that diaphoresis, excited by bother or massage, improves digestion, but that produced by pilocarpine retards it. He recommends the Russian bath and massage in the treatment of some forms of dyspepsia.

---

During the recent French invasion of Tonquin, the surgeons attempted to employ strict antisepsis in military surgery. The soldiers were furnished with antisepsis tampons for the immediate occlusion of wounds, a procedure which was found to be impracticable in the majority of cases. Carbolized sheets were used for covering the patients until they could be brought to the field hospital, where the wounds were washed and dressed antiseptically, and the patient left until operations, if necessary, could be performed. While the antisepsis methods succeeded in reducing the mortality among the wounded, the results obtained did not equal those of Reyher in the Russo-Turkish war.

